# Automated extraction of Biomarker information from pathology reports

**DOI:** 10.1186/s12911-018-0609-7

**Published:** 2018-05-21

**Authors:** Jeongeun Lee, Hyun-Je Song, Eunsil Yoon, Seong-Bae Park, Sung-Hye Park, Jeong-Wook Seo, Peom Park, Jinwook Choi

**Affiliations:** 1 Interdisciplinary Program for Bioengineering, Graduate School, Seoul National Universty, Seoul, Republic of Korea; 2 School of Computer Science and Engineering, Kyungpook National University, Daegu, Republic of Korea; 3 PAS1 team, TmaxSoft, Gyeonggi-do, Republic of Korea; 4 Department of Pathology, College of Medicine, Seoul National University, Seoul, Republic of Korea; 5 Department of Biomedical Engineering, College of Medicine, Seoul National University, Seoul, Republic of Korea; 6 Department of Industrial Engineering, Ajou University, Suwon, Republic of Korea

**Keywords:** Biomarkers, Cancer disease knowledge representation model, Pathology reports, Natural language processing, Clinical decision-making

## Abstract

**Background:**

Pathology reports are written in free-text form, which precludes efficient data gathering. We aimed to overcome this limitation and design an automated system for extracting biomarker profiles from accumulated pathology reports.

**Methods:**

We designed a new data model for representing biomarker knowledge. The automated system parses immunohistochemistry reports based on a “slide paragraph” unit defined as a set of immunohistochemistry findings obtained for the same tissue slide. Pathology reports are parsed using context-free grammar for immunohistochemistry, and using a tree-like structure for surgical pathology. The performance of the approach was validated on manually annotated pathology reports of 100 randomly selected patients managed at Seoul National University Hospital.

**Results:**

High F-scores were obtained for parsing biomarker name and corresponding test results (0.999 and 0.998, respectively) from the immunohistochemistry reports, compared to relatively poor performance for parsing surgical pathology findings. However, applying the proposed approach to our single-center dataset revealed information on 221 unique biomarkers, which represents a richer result than biomarker profiles obtained based on the published literature. Owing to the data representation model, the proposed approach can associate biomarker profiles extracted from an immunohistochemistry report with corresponding pathology findings listed in one or more surgical pathology reports. Term variations are resolved by normalization to corresponding preferred terms determined by expanded dictionary look-up and text similarity-based search.

**Conclusions:**

Our proposed approach for biomarker data extraction addresses key limitations regarding data representation and can handle reports prepared in the clinical setting, which often contain incomplete sentences, typographical errors, and inconsistent formatting.

**Electronic supplementary material:**

The online version of this article (10.1186/s12911-018-0609-7) contains supplementary material, which is available to authorized users.

## Background

Precision medicine is a newly emerging trend in medicine, whereby individualized medical treatments are designed based on the specific biologic information of each patient. Traditionally, tumors are classified based on their histological features. However, in the era of precision medicine, the biological behavior is interpreted in terms of not only histological morphology but also genomic features of the tumor.

For individualized diagnosis, pathologists currently rely substantially on immunohistochemistry (IHC) findings, which provide clues regarding genomic features in terms of relevant biomarkers [1]. Biomarkers reflect the biologic behavior of tumors, including pathogenicity and pharmacologic response [2, 3]. Thus, to ensure a reliable histologic diagnosis, it is important to have access to statistical data from pathology reports regarding similar patients, which can be achieved through the retrospective study of relevant reports describing biomarker findings, tumor staging, and morphologic features of the cancer [3–6].

Pathpedia (www.pathpedia.com) [7] is an online resource of pathology information providing statistical data including biomarker levels, anatomical pathology features, and clinical pathology features. The main advantages of Pathpedia include high-level, manual curation of data and the large number of information sources (up to 4000 references). However, there are disadvantages to using journal articles as the main source of reference data. Specifically, certain biomarkers are discussed in a limited number of journal articles, which precludes data verification. Moreover, there is limited coverage of various ethnic groups and uneven data distribution regarding race, life patterns, nutritional habits, geology, and climate, which precludes genomic-level comparisons based on data from Pathpedia.

The present study focused on the Korean population and involved careful review of 82,291 pathology reports, including IHC and surgical pathology (SP) reports, from Seoul National University Hospital (SNUH). In order to facilitate the detection of potential relationships between various immunologic biomarkers and pathologic diagnosis, we previously developed a web-based information system [8] designed to compute and display statistics of clinical data extracted from pathology reports (e.g., affected organ, diagnosis, cancer staging information, and IHC findings).

We further describe related work in the field of automated extraction of data from medical reports, and then introduce our information extraction approach specifically tailored to pathology reports. We then describe in detail the result of text processing via our proposed approach, as well as the relevance of the findings. Finally, we cover the limitations and implications of our proposed solution.

Most previous studies on automatic extraction of biomarker data focused on biomedical literature [9–11]. Yonesi et al. [9] suggested a system for improved recognition of biomarker names in published literature, which is implemented in ProMiner [10] and represents a rule-based system for gene name normalization. The BioNER [11] system uses a custom-made biomarker-specific disease dictionary to extract disease-related biomarkers from MEDLINE publications. However, the performance of these systems is insufficient for processing pathology documents because of intrinsic differences between such clinical documents and published scientific articles. Specifically, to enhance readability in the clinical setting, pathology reports generally do not follow formal grammar rules and are instead written in a keyword-oriented style and formatted using many line breaks, white spaces, and hyphens. This type of formatting makes it difficult to automatically detect the sentence boundary and apply normalization of term variation, which is particularly relevant because current solutions for biomarker data mining employ context to a substantial extent.

Although some studies have attempted to extract biomarker information, these typically focused on extracting cancer-related information (e.g., histologic type and stage) from pathology reports, and, in particular, from SP reports. Such studies [12–20] typically apply pattern-based natural language processing for extracting information on specific types of cancer (especially breast or lung cancer), which is not applicable to mining data on other types of cancer. On the other hand, Coden et al. [19] proposed a pan-cancer knowledge representation model for data from SP documents. Specifically, the Cancer Disease Knowledge Representation Model (CDKRM) defines what entities can be extracted and what relations these entities can have with each other. However, the CDKRM does not cover IHC findings and does not define how to combine biomarker information parsed from IHC reports with corresponding pathologic findings parsed from SP reports. For the same reason, the PEP [20] system, which follows the CDKRM for information extraction, cannot extract biomarker information by default and also requires modification to handle keyword-oriented SP reports typically encountered in clinical practice.

Unlike biomedical literature, clinical reports sometimes contain specific jargons and spelling errors [21], and therefore the identified medical terms require further normalization, i.e., ensuring that clinical terms with the same meaning are represented in the model using the same term. The cTAKES approach [22] is well known for its ability to recognize most medical terms (including anatomical sites and disease names) from clinical documents and to correctly normalize these terms to their Unified Medical Language System (UMLS) [23] identifiers. However, UMLS does not cover all variants of biomarker-related terminology used in IHC. Even though cTAKES can employ other dictionaries than UMLS for normalization, as it was designed to handle general clinical documents following formal English grammar, it is difficult to customize cTAKES for efficient normalization of keyword-based reports such as IHC.

## Methods

### Representation of biomarker knowledge

**Fig. 1:**
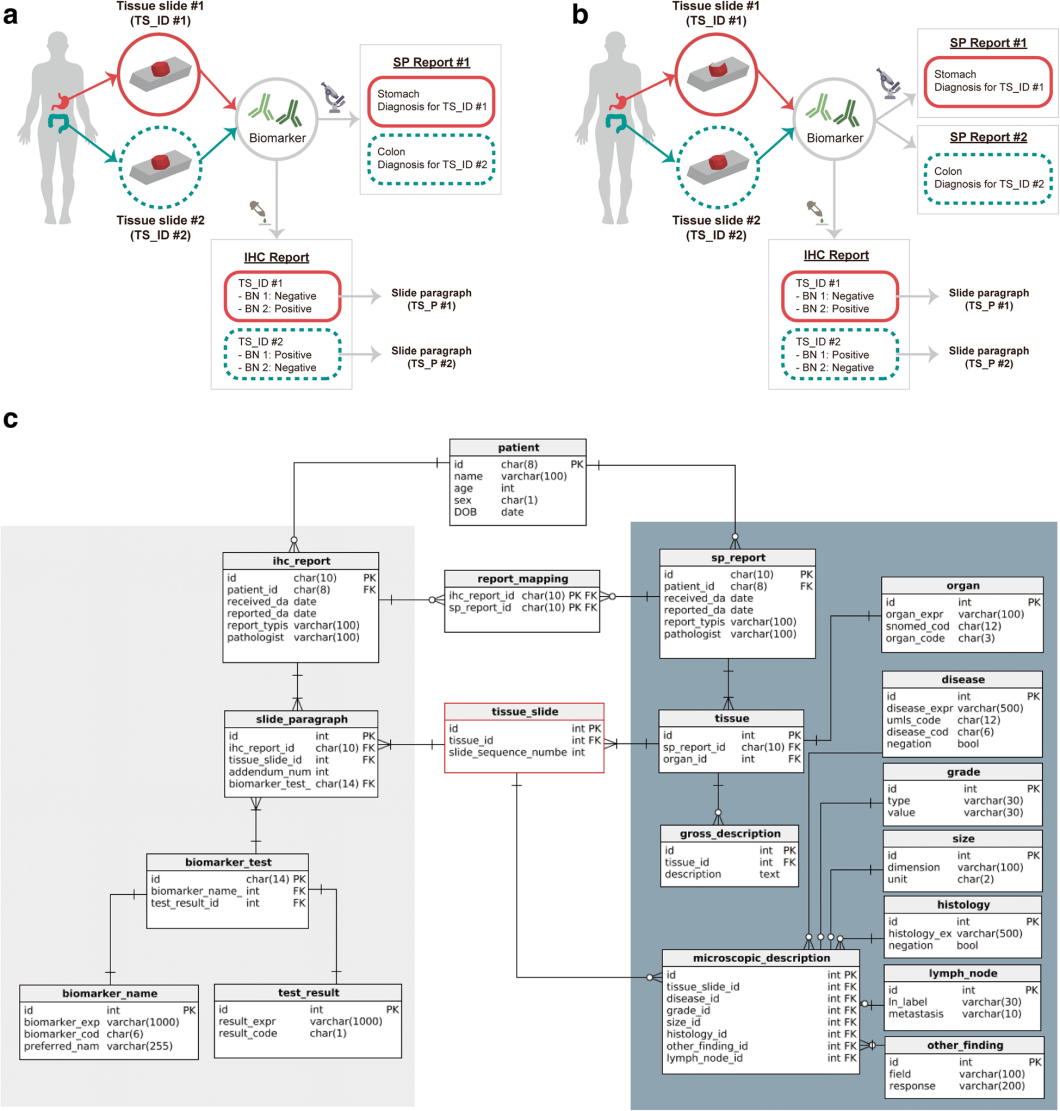
Representation model for information extraction of biomarker. **a** Reporting system for multi biopsy samples from a patient. As same biomarker test can be conducted for tissue slide #1 and tissue slide #2 of the patient, a IHC report can contain multiple “slide paragraph (TS_P)”. Also, multiple pathologic findings derived from multiple IHC tests can be reported in one SP report **b**) or in separate SP reports. **c** Representation model for information extraction of biomarker from pathology reports in Seoul National University Hospital. IHC:immunohistochemistry, SP:surgical pathologic, BN:biomarker name

Our data model for representing biomarker knowledge is similar to the CDKRM [19], but it contains a new framework for handling IHC reports. Specifically, the CDKRM covers only microscopic pathologic findings from SP reports as model entities, whereas the model developed in the present study can represent biomarker test results extracted from IHC reports. Additionally, our model has the ability to define which biomarker test described in an IHC report represents evidence for which microscopic finding described in an SP report, which helps avoid incorrect assignment of IHC findings to pathology findings described in the SP report of another patient or of the same patient but for a different indication. For example, when the patient shows symptoms suggesting metastatic cancer, the physician may conduct the same IHC test for several tissue slides obtained from the same patient (Fig. 1a and b, Additional file 1: Figure S1a and S1b). The results of such an IHC test may be reported either in an addendum to the original IHC and SP reports (Fig. 1a), or in an entirely new report (Fig. 1b, Additional file 1: Figure S1a and S1b) [24]. Thus, when designing the data representation model, it is important to take into account that the patient may have one or more SP reports with various microscopic findings for different tissue slides.

To facilitate appropriate association between the findings described in IHC reports and those described in SP reports, we assumed that a tissue slide represents the minimum unit for describing a pathologic finding. This assumption is based on the fact that almost every IHC report states the serial number of the target tissue slide at the top of the results of each analysis performed as the guidelines of College of American Pathologist (CAP) (Additional file 1: Figure S1c) [25, 26]. Furthermore, some descriptions of pathologic findings in the SP report explicitly state which tissue slide provides evidence for that particular finding. Thus, we associated IHC findings with microscopic findings based on tissue slide identifiers (TS_IDs), which represent the serial numbers of tissue slides; furthermore, we introduced the tissue slide paragraph (TS_P) to refer to a set of IHC findings corresponding to a single tissue slide.

Parsing IHC reports using TS_P provides another advantage for cases where the same IHC tests are repeated within a certain time interval because previous tests showed unstable results (Additional file 1: Figure S1a and S1b). In such situations, the same tissue slide (i.e., TS_ID) will show different IHC results, which can be traced easily by collecting all TS_Ps pointing to the same TS_ID, and subsequently checking the time stamps.

However, parsing the SP reports according to this model can be challenging. Most SP reports at SNUH describe microscopic findings organized by the anatomical site of the tested tissue sample, not by TS_ID which is consistent with guidelines of CAP (Additional file 2: Figure S2b and S2c) [24]. Therefore, when the SP report describes the microscopic findings in tissue samples from more than two different anatomical sites or in samples of the same tissue but with distinct pathological characteristics, it is not appropriate to simply map all tissue slides to the anatomical site first mentioned in the SP report. Instead, the system needs to determine the anatomical site of origin for each tissue slide. Fortunately, since one tissue sample is typically resected from each anatomical site, the algorithm steps for determining the anatomical site need to be triggered only when the SP report mentions more than two anatomical sites, thereby minimizing the computational burden of this procedure. A scheme describing our data representation model is given in Fig. 1c.

### Information extraction from IHC reports

**Fig. 2:**
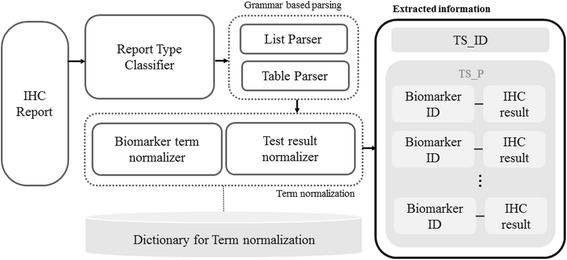
Flow chart of information extraction from IHC report with an example. Step #1) Classify type of IHC report and choose appropriate parser for the input. Step #2) Normalize biomarker names recognized from step #1 using BN dictionary. Step #3) Normalize test results recognized from step #1 using TR dictionary. BN: biomarker name, TR: test result

SNUH reports adopt a semi-structured style for describing IHC findings, further classified as list- or table-based style (Additional file 1: Figure S1a and S1b). We designed the information extraction system in consideration of this semi-structured style, to ensure simplicity and accuracy of parsing. After collecting a set of patterns for each style (i.e., list- or table-based) from training set, we defined a context-free grammar to identify which tokens stand for biomarker names (BNs; e.g., p53, p63, Cyclin-D1) and which tokens represent the corresponding test results (TRs; e.g., positive, negative). The parsing also takes note of the boundary of each TS_P tagged with the appropriate TS_ID. Parsing is achieved using either a list parser or a table parser (Fig. 2), and items parsed from IHC reports are associated with corresponding microscopic findings parsed from SP reports based on TS_ID.

Biomarker term normalization handles terminology variation in BNs and ensures that, for example, ‘Wilms’ tumor 1 protein’, ‘WT-1′, ‘Wilms tumor 1-protein’, and ‘WT1 (4)’ are converted into the single representative form ‘Genes, Wilms Tumor’. To facilitate term normalization, we created a BN dictionary for dictionary look-up and text similarity-based search. The dictionary is based on a list of official product names of biomarkers used at SNUH, comprising 184 original BNs, which we annotated with Preferred Terms (P_BN) of Medical Subject Headings (MeSH) codes. If there was no MeSH entry for a given BN, the P_BN assigned by the pathologists was used as the general term. The list of BNs was then automatically expanded with a set of hand-crafted rules to cover simple variations in notation, such as addition of hyphens or white spaces. This type of dictionary expansion was designed to make text similarity-based searching fast and efficient.

The rules applied to expand the BN dictionary (Table 1) are similar to those described by Bravo et al. [11] for term normalization of gene/biomarker protein names in the biomedical literature, except we remove laboratory-related terms included in parenthesis, so that ‘WT-1 (4)’ and ‘WT-1 (Repeat)’ are normalized to ‘WT-1’. Consequently, the number of entries in the expanded BN dictionary was 3370. This BN dictionary was cross-checked by three pathologists at SNUH.

**Table 1:** Term expansion rules for biomarker names

Rule	Example
1) Replace Roman numbers with the corresponding Arabic numbers	Factor VIII → Factor 8
2) Replace Greek symbol with the corresponding letters	CD79 α → CD79 alpha
3) Remove laboratory related terms	CD10(repeat) → CD10
4) Generate new variants	GAL3 = GAL-3 = GAL 3
5) Convert to lowercase	DESMIN→ desmin

Term variations in TRs were also considered. For example, ‘positive in mesothelial cells’ and ‘positive in tumor cells’ are normalized to ‘Positive’, whereas and ‘focal weak positive’ and ‘a few positive cells’ are normalized to ‘Focal positive’. Unfortunately, as TR normalization requires background knowledge about the tested biomarkers, it was not feasible to create a TR dictionary by applying the same rule-based approach as that used for creating the BN dictionary. Therefore, three pathologists manually created a TR dictionary based on all TRs from training set to assign the normalized TR terms: “Positive”, “Focal Positive”, “Negative”, or “Error” (i.e., failed IHC test or spelling error that precludes normalization of the detected TRs). This TR dictionary was then used for normalization of TR terms by dictionary look-up.

### Information extraction from SP reports

**Fig. 3:**
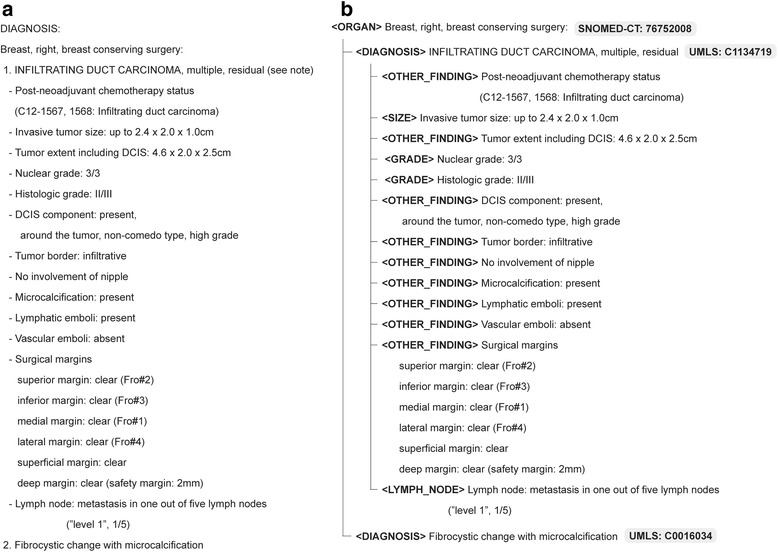
**a** Excerpt of a SP report, **b** corresponding parsing tree of SP report in 3a

SP reports are parsed and processed in a similar manner as that employed for handling IHC reports. However, unlike IHC reports, SP reports are rather unstructured in terms of reporting style, and thus, the grammar-based parsing approach used for parsing IHC reports was not adequate for parsing SP reports, as it could not account for all patterns of reporting styles employed in SP reports. Fortunately, in most SP reports archived at SNUH, the first few lines typically mention what organ the tissue sample originated from, whereas the subsequent lines describe the pathologic findings for that tissue [24]. Additionally, when pathologic findings are reported for a specific disease, these findings are grouped under the disease name with an indentation which is consistent with recommendation of CAP for visual separation (Additional file 2: Figure S2b and S2c) [24]. We took this behavioral tendency into account when designing the parser, aiming to reduce the search space for each item and improve parsing accuracy. Specifically, we implemented a tree-based parsing approach of the SP reports, whereby the tree root node is designated in terms of the organ name. In the example illustrated in Fig. 3, the organ name “Breast” appears on the first line of the SP report, so the system creates a tree with the root node containing the first line. Because the next line is numbered and starts with the disease name “1. INFILTRATING DUCT CARCINOMA, multiple, residual (see note)”, this line is assigned as the first sub-node of the root node, at the same level as other numbered lines, such as “2. Fibrocystic change with microcalcification”. Because the lines below the first sub-node have indentations, the system assigns them as sub-nodes of the first sub-node. In this way, the system can generate candidate boundaries for organs, diseases, and specific pathologic findings.

In other words, to create a tree with the root node represented by the organ, the parser must recognize the organ name correctly. To implement such a parser, we first created a dictionary based on terms corresponding to organ names from the Systematized Nomenclature of Medicine-Clinical Terms (SNOMED-CT). The system performs a dictionary look-up operation and calculates a look-up score as the linear sum of the Edit distance, string kernel, Jaro-Winkler distance, and soft term frequency-inverse document frequency statistic. If the look-up score is above a certain threshold, the system registers the term into the organ terminology database, which consists of a normalized set of terms describing organs. We used a training set of SP reports to build the organ terminology database according to this method, which was then verified and curated by two clinicians at SNUH. When parsing the SP reports, the system applies a similar approach to recognize and normalize disease names with UMLS disorder names. If a node corresponding to a disease name is found to have a sub-tree, all its sub-nodes are considered to represent detailed microscopic findings regarding that disease. Finally, for recognition of negation and metastasis terms, we used a set of patterns manually created based on the same training set of SP reports.

### Merging IHC and SP information

Biomarker information extracted from IHC reports and corresponding microscopic findings extracted from SP reports are matched according to TS_P and TS_ID.

### Datasets and performance evaluation

We obtained 41,772 IHC reports and 40,519 SP reports archived at SNUH between 2007 and 2012 (Table 2). Subsequently, reports created between 2007 and 2011 were used as the training data set, whereas later reports were used as a validation data set. Additionally, in order to create a gold standard for the evaluation, we randomly selected 400 patients for whom we manually annotated the reports (508 IHC reports and the corresponding 831 SP reports) using the BRAT tool [27]. There were three trained annotators. Each document of patient was annotated by two annotators for normalization of BNs and TRs along with recognition of boundaries of entity mentions. Inter-annotator agreement was measured by F-measure [28]. (Additional file 3: Table S1).

**Table 2:** Statistics of SNUH dataset

Type of report	2007	2008	2009	2010	2011	2012	Total
IHC	0	6370	7611	8067	10,889	8830	41,772
SP	160	6338	7507	7893	10,341	8280	40,519

*IHC* Immunohistochemistry reports, *SP* Surgical pathology reports

For validation, we selected only those IHC reports for which the corresponding SP reports described information regarding only one organ and one diagnosis, so as to assess solely the issue of data extraction itself. The issue of discriminating findings that refer to multiple organs or diagnoses listed in the SP report will be covered in future work, as this aspect also involves the processing of SP report data without corresponding IHC findings (e.g., findings of the gross examination).

The training data was mainly used to generate patterns while making hand-crafted rules for parsing. Also, we used the training set to manually curate the dictionaries for normalization of BNs and TRs. Validation of information extraction was performed on the gold standard set we manually made.

## Results

The IHC report parser extracts TS_ID, BN, and corresponding TRs. The ability of the parser to recognize the boundaries of these entities was evaluated by exact matching, which indicated that the parser recognized the entity boundaries well, with F-1 scores of 1, 0.998, and 0.9978 for TS_ID, BN and TR, respectively (Table 3). After parsing each entity, the system normalized term variants to a single representative term, which was also achieved with high performance, namely with F-1 scores of 0.972 and 0.969 for BN and TR, respectively.

**Table 3:** Extraction performance for IHC and SP

A	Recognition			Normalization		
	Recall	Precision	F1	Recall	Precision	F1
TS_ID	1	1	1	–	–	–
BN	0.999	1	0.999	1.000	0.946	0.972
TR	0.998	0.998	0.998	1.000	0.939	0.969
B	Exact Matching					
	Recall	Precision	F1			
Organ	0.896	0.953	0.924			
Diagnosis	0.794	0.427	0.556			
C	Overlap matching					
	Recall	Precision	F1			
Organ	0.901	0.961	0.930			
Diagnosis	0.794	0.754	0.773			

*TS_ID* Tissue slide ID, *BN* Biomarker name, *TR* Test result

On the other hand, the SP report parser was evaluated only in terms of term boundary recognition for organ and diagnosis, as the data set used for training only contained such information. The exact matching test indicated good performance for organ name recognition but poor performance for diagnosis recognition (Table 3). Because the boundaries of the disease term showed some differences between annotators, we also applied overlap matching to evaluate the performance of the parser regarding term boundary recognition. Although the exact matching test results indicated low performance (F-1 score 0.556), the overlap matching test results indicated higher performance (F1 score 0.773).

When applying the developed system to all pathology reports (i.e., including the reports used as a validation data set), 45,999 TS_Ps were found within 41,765 IHC reports. The remaining 7 IHC reports could not be parsed because they did not include TS_ID. Furthermore, 4135 of 45,999 TS_Ps identified were ignored because their corresponding SP reports were not included in our target dataset. Within the 41,864 TS_Ps analyzed, the system recognized 206,534 BNs and successfully normalized 205,759 BNs into 215 P_BNs. Each extracted BN was associated with a corresponding TR. All but 105 TRs were correctly normalized. Finally, we obtained 205,627 BN-TR pairs.

**Fig. 4:**
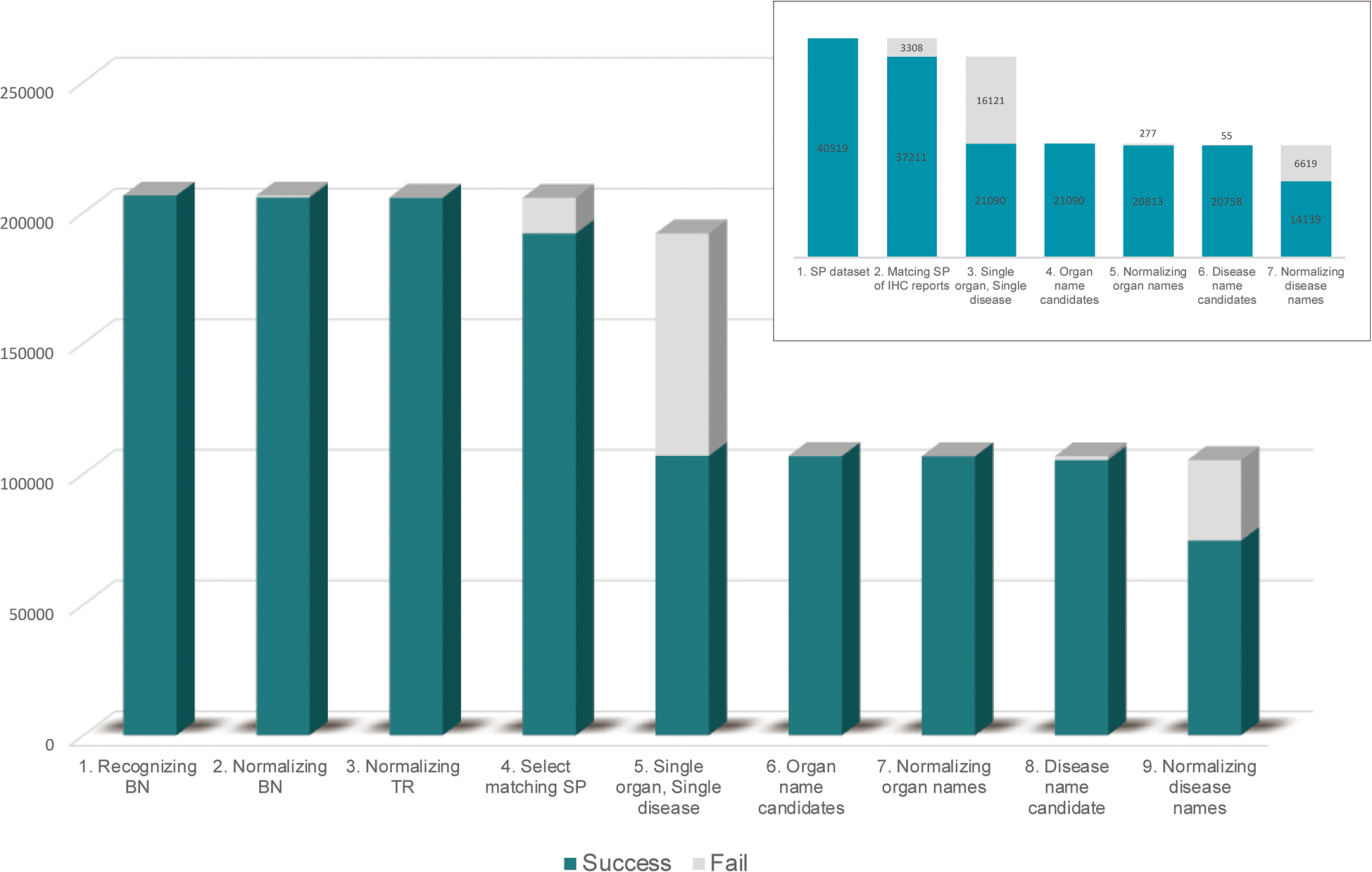
The process of parsing SP reports; the main graph showing statistics for total BNs, and the corner graph showing statistics for total SP reports

When applying the system to parse 37,211 SP reports corresponding to 41,864 TS_Ps extracted from IHC reports (Fig. 4), a total of 5012 SP reports were found to describe no microscopic diagnosis. Furthermore, only SP reports mentioning a single organ and a single diagnosis were retained (21,090 SP reports), within which 21,090 candidates for organ names were recognized. With the exception of 277 organ name candidates, all other candidates were successfully normalized into 46 representative organ names. Subsequent parsing of disease names resulted in 20,758 candidate disease names, of which 68.11% were successfully normalized. The reason for this relatively lower success rate of the normalization is likely related to including inappropriate candidates of disease names, as well as to the high term variation for disease names. The process of parsing SP reports is illustrated in Fig. 4, with the main graph showing statistics for total BNs, and the corner graph showing statistics for total SP reports.

**Fig. 5:**
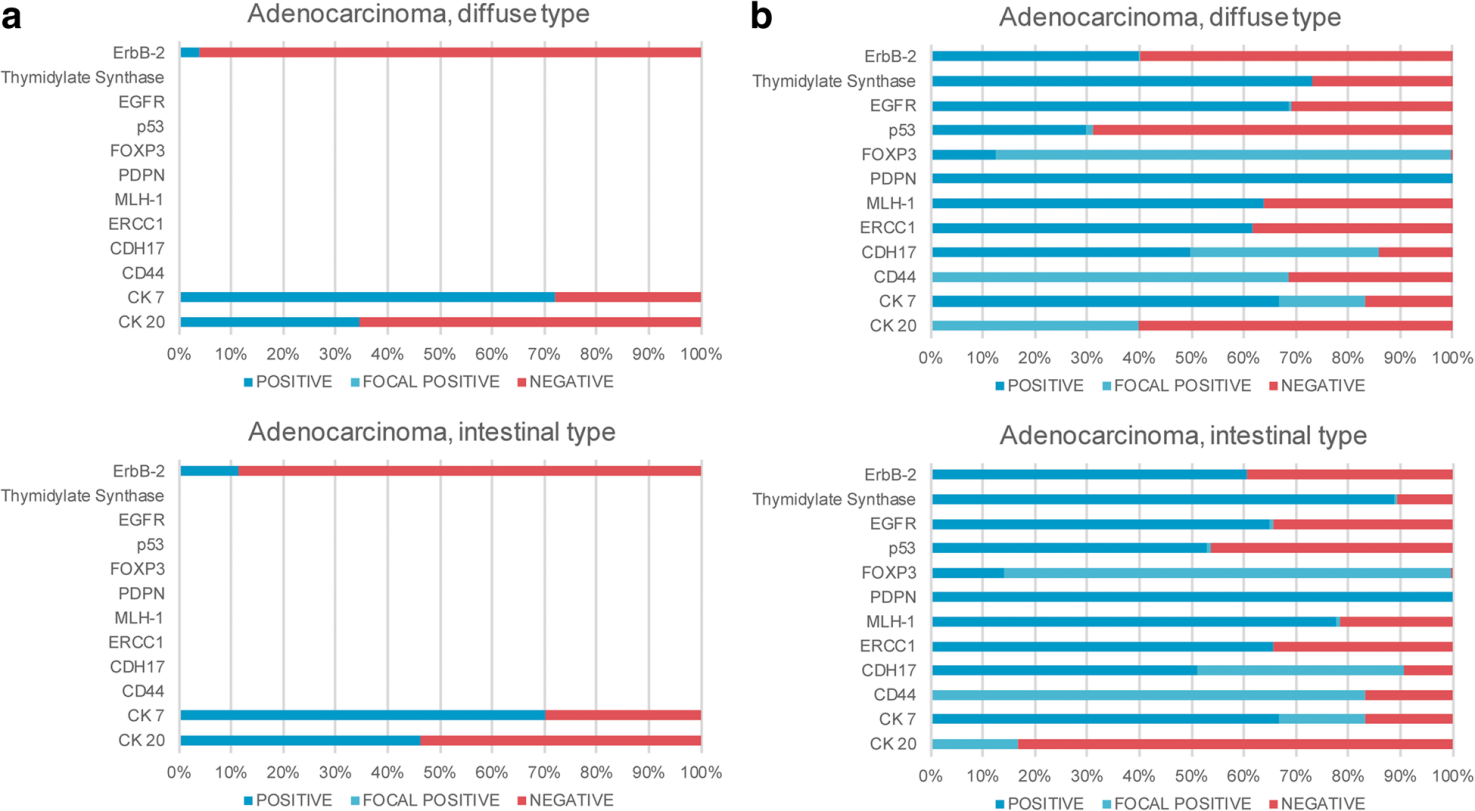
Comparison of the statistics provided by our proposed approach for information extraction with the statistics provided by Pathpedia regarding the positive rate of biomarker assays. **a** statistics provided by Pathpedia, **b** the statistics provided by our proposed approach for adenocarcinoma, diffuse type and intestinal type in stomach

Finally, we compared the statistics provided by our proposed approach for information extraction with the statistics provided by Pathpedia (Fig. 5) regarding the positive rate of biomarker assays. The first important difference between the SNUH- and Pathpedia-based findings is the composition of the set of biomarkers employed for such analyses. For example, the mean number of assay attempts for ErbB-2, thymidylate synthase, EGFR, p53, FOXP3, PDPN, MLH-1, ERCC1, CDH17, CD44, CK 7, and CK 20 is 469 based on SNUH data, compared to only 132 based on Pathpedia data. Moreover, although the number of ErbB-2 assays was similar (720 and 650 assays for SNUH and Pathpedia, respectively), the rate of positives (including focal positive) was substantially different, with 290/720 for SNUH and 26/650 for Pathpedia.

## Discussion

The proposed approach for automated extraction of biomarker data showed high performance for parsing IHC reports (very high F1-scores), which is due to the very stable reporting format used at SNUH. Specifically, since 2008, SNUH pathologists have written IHC reports using one of only two reporting formats (i.e., list- or table-based). Although there were spelling errors in writing, detecting certain formatting determinants such as semi-colon (“;”), colon (“:”), or vertical bar (“|”) was extremely helpful in accounting for report format variations while still using relatively simple grammar; in fact, we were able to update the internal database of IHC report templates with a new version.

**Fig. 6:**
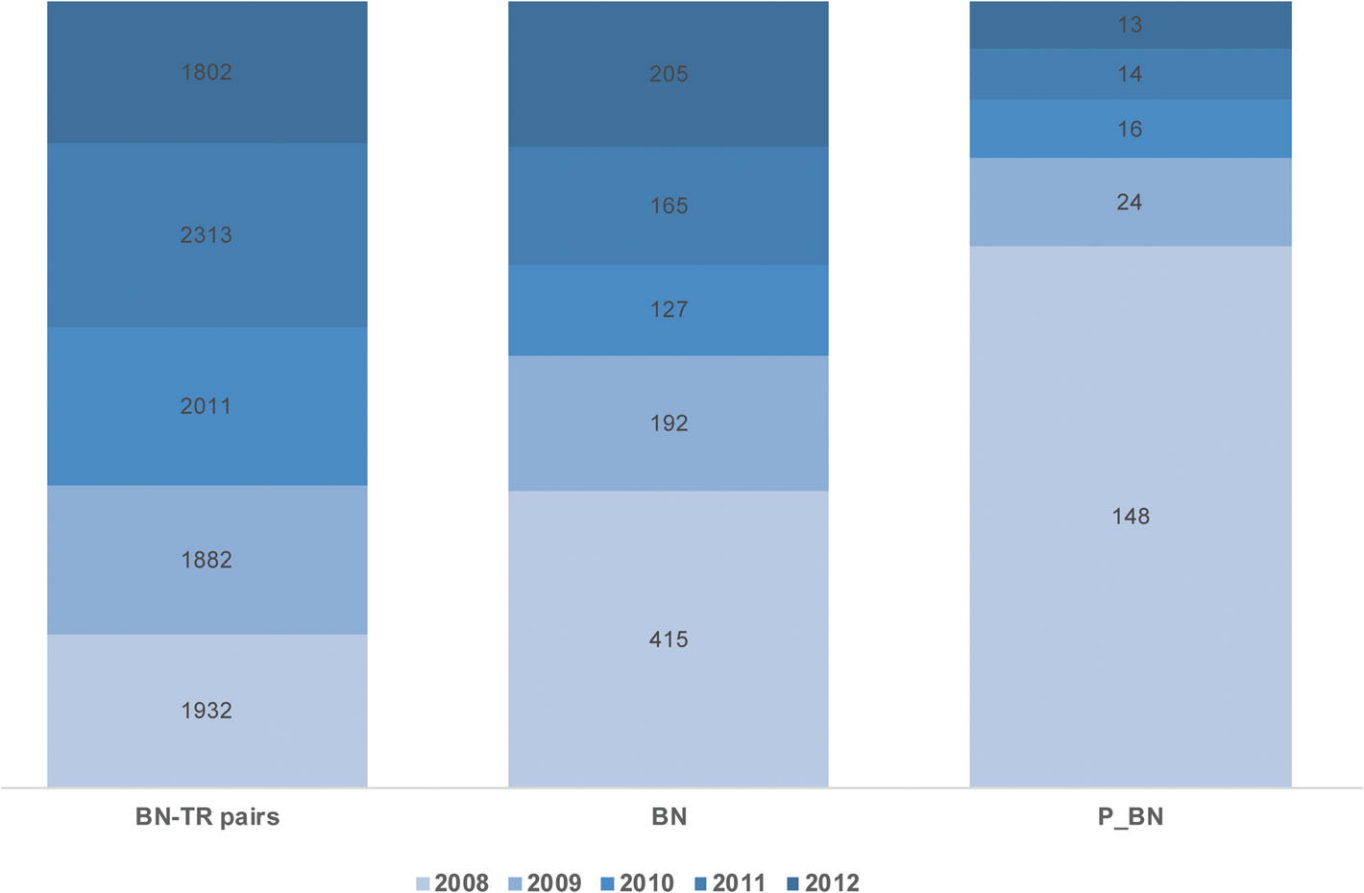
The creation rate of new BN term variants per year. Although a similar number of new IHC tests (described as BN-TR pairs) were performed each year at SNUH, the number of new biomarker name (BN) variants increased steadily every year. This increase is partially related to the increase of a new type of biomarkers (described as P_BN) which SNUH analyzes

Our approach involves using dictionaries for recognizing and normalizing BNs and TRs, which we created based on initial processing of the training data set. As depicted in Fig. 6, a similar number of new IHC tests (described as BN-TR pairs) were performed each year at SNUH, and the number of BN terms increased steadily every year. This finding is related to the fact that, every year, SNUH purchased a new type of biomarker. Moreover, SNUH received and archived IHC reports prepared by pathologists at other hospitals regarding patients referred to SNUH for consultation. Fortunately, most variations were covered by the expanded BN dictionary, but terms related to the new biomarkers had to undergo normalization according to UMLS terminology. Notably, there was substantially higher variation in TR terminology, and the parser required almost ten times longer to recognize and normalize TRs than to do so for BNs. Moreover, as some BNs have numeric TRs whose ranges may differ with cell type, classifying the TRs into four categories (positive, focal positive, negative, and error) resulted in some controversy between consulting pathologists.

Unlike IHC reports, SP reports at SNUH showed high variability in reporting style and format, and creating the SP report parsing tree with a single organ root node was sometimes difficult because some SP reports contained a description of findings of the macroscopic or frozen section evaluations, which are commonly performed during surgery. When pathologists deem that further tests are necessary or they lack sufficient evidence for a definite diagnosis, they report several candidate disease names. As the goal of extracting IHC and SP data in this manner is to facilitate detection of potential relationships between IHC profiles and pathologic diagnosis, further study is warranted to develop an automated framework for discriminating between final and intermediate diagnosis. Here, we chose to include in the validation data set only SP reports describing a single sample with a single, explicitly stated diagnosis.

Parsing the SP reports produced the name of the organ of origin of the tissue sample, and the diagnosis based on microscopic findings. Under the assumption that the pathologic findings are described after the organ is mentioned, we could discriminate the lines describing the organ of origin from the lines describing the disease, as well as from the lines describing the actual findings, and organized this information into a tree-like structure. We then used dictionaries based on SNOMED-CT and UMLS to further normalize the recognized organ names and disease names, but these dictionaries did not cover all parts of the disease or organ names. For example, because UMLS had an entry only for “gastric carcinoma”, whereas the SP report was found to refer to “multiple gastric carcinoma” or “early gastric carcinoma”, we normalized both disease terms to the same identifier. By recording the entire line containing the disease name (part of which likely corresponds to at least one UMLS term), we aimed to preserve as much information as possible.

Because we have parsed IHC and SP reports based on assumptions that may apply only to reports from SNUH, our approach may not be as easily applied to other hospital data. As we checked two branch hospitals of SNUH which operate independent hospital information system, both of which adopt the closely matched reporting format with SNUH. It is likely that this result comes from a policy that adheres to guidelines of CAP that recommend to display multiple diagnostic findings with visual separation such as an indentation for indicating a subordinate relationship or (checklist item: response) style [24]. Thus, if a hospital is referring to guidelines of CAP, the representation model we propose would be reasonable choice when localization efforts for rule-based parsing such as adjusting formatting determinants for visual separation could be made.

In this study, we proposed a representation model and developed the system from scratch to apply the model for information extraction of SNUH pathology reports. Although our parsing approach is not remarkably innovative, we could get data that reveals the differences between the Korean population and the other ethnic group of literature, as we defined the entity-relationship model for reliable associations between IHC results and microscopic findings and developed a systematic strategy. Also, in addition to that the web-based information system which calculates multi-conditional statistics of the extracted data from this study showed stable performance, 25 pathologists confirmed that the statistics is reliable in the previous study [8]. Accordingly, we could say that our representation model is reasonable for presenting the entities for pathology reports.

## Conclusion

We proposed a systematic approach to mine and corroborate biomarker information from IHC reports and SP reports. According to our new data representation model for biomarker knowledge from pathology records, IHC reports are parsed using a grammar-based approach, whereas the corresponding SP reports are parsed using a tree-based approach. Term variations are resolved by normalization to corresponding preferred terms determined by expanded dictionary look-up and text similarity-based search. Finally, IHC data and relevant pathological findings are associated with corresponding clinical information from SP reports based on TS_ID and TS_P. When tested on a manually annotated dataset, acceptable F-scores were obtained for parsing (with term recognition and normalization) of both IHC reports and SP reports. We applied the developed approach to process 41,772 IHC reports and 40,519 SP reports archived between 2007 and 2012 at SNUH, and obtained information on 221 unique biomarkers, which is consistent with biomarker profiles obtained from the literature but provides more comprehensive statistics regarding biomarker use.

## Additional files

Additional file 1: **Figure S1.** a) list-style of IHC report of a patient, b) table-style of IHC report of the same patient reported later in a separate document, c) excerpt of synoptic report template from College of American Pathologists (CAP) which shows that tissue block number should be described on the IHC report. (TIF 898 kb)

Additional file 2: **Figure S2.** Comparison of SP report styles between SNUH and report templates from CAP. a) SP report at SNUH which shows the combination of synoptic report style and non-synoptic report style. b) synoptic report template for ductal carcinoma in situ of the breast which presents organ information on the first line and describes microscopic findings in the following lines. The synoptic report template shows the grouping of pathologic findings using indentation for better visual separation. c) non-synoptic report template provided by CAP. The first line contains organ information and the second line contains the diagnosis information with indentation for visual separation. (TIF 985 kb)

Additional file 3: **Table S1.** Inter-Annotator Agreement (IAA) for gold standard set. (XLSX 8 kb)

## Abbreviations

BN: Biomarker Names
BRAT: Brat Rapid Annotation Tool (developed by Stenetorp et al.)
CAP: College of American Pathology
CDKRM: Cancer Disease Knowledge Representation Model (a knowledge model proposed by Coden et al.)
cTAKES: Clinical Text Analysis Knowledge Extraction System (an apache based open source natural language system for information extraction from EMR)
IHC: Immunohistochemistry
P_BN: Preferred term of the Biomarker Name in MeSH
PEP: Pathology Extraction Pipeline (developed by Ashish et al.)
SNOMED: Systematized Nomenclature of Medicine Clinical Terms
SNUH: Seoul National University Hospital
SP: Surgical Pathology
TR: Test Result
TS_ID: Tissue Slide Identifier
TS_P: Tissue Slide Paragraph
UMLS: Unified Medical Language System
WT-1: Wilms’ Tumor 1 protein
