# Supplementary figures and images for: Automated extraction of Biomarker information from pathology reports
Source: BMC Med Inform Decis Mak. 2018 May 21;18:29. doi: 10.1186/s12911-018-0609-7 (PMC5963015; doi:10.1186/s12911-018-0609-7)

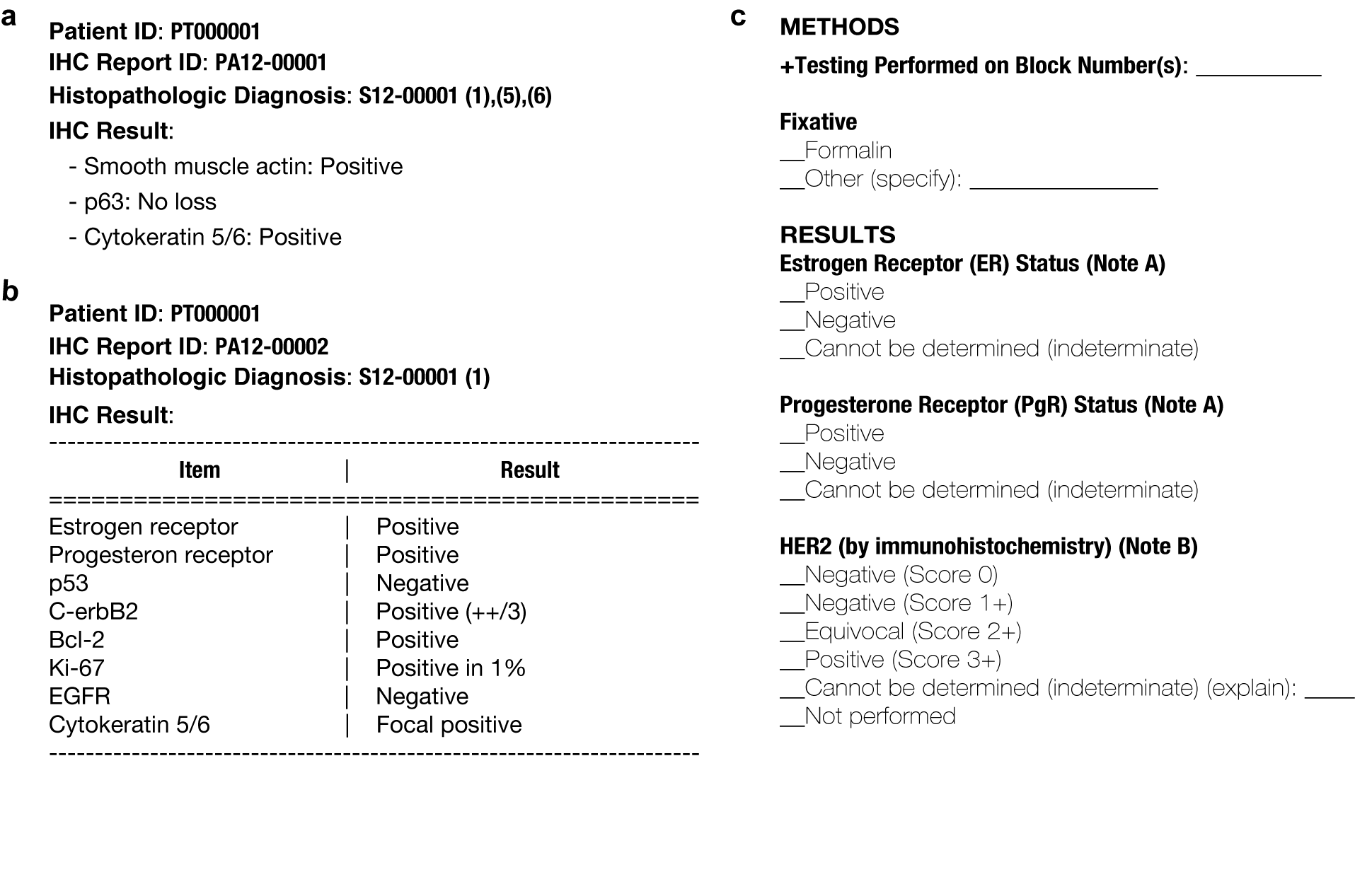

Supplement: Supplementary file 1 — Figure S1. a) list-style of IHC report of a patient, b) table-style of IHC report of the same patient reported later in a separate document, c) excerpt of synoptic report template from College of American Pathologists (CAP) which shows that tissue block number should be described on the IHC report. (TIF 898 kb) [file 12911_2018_609_MOESM1_ESM.tif]

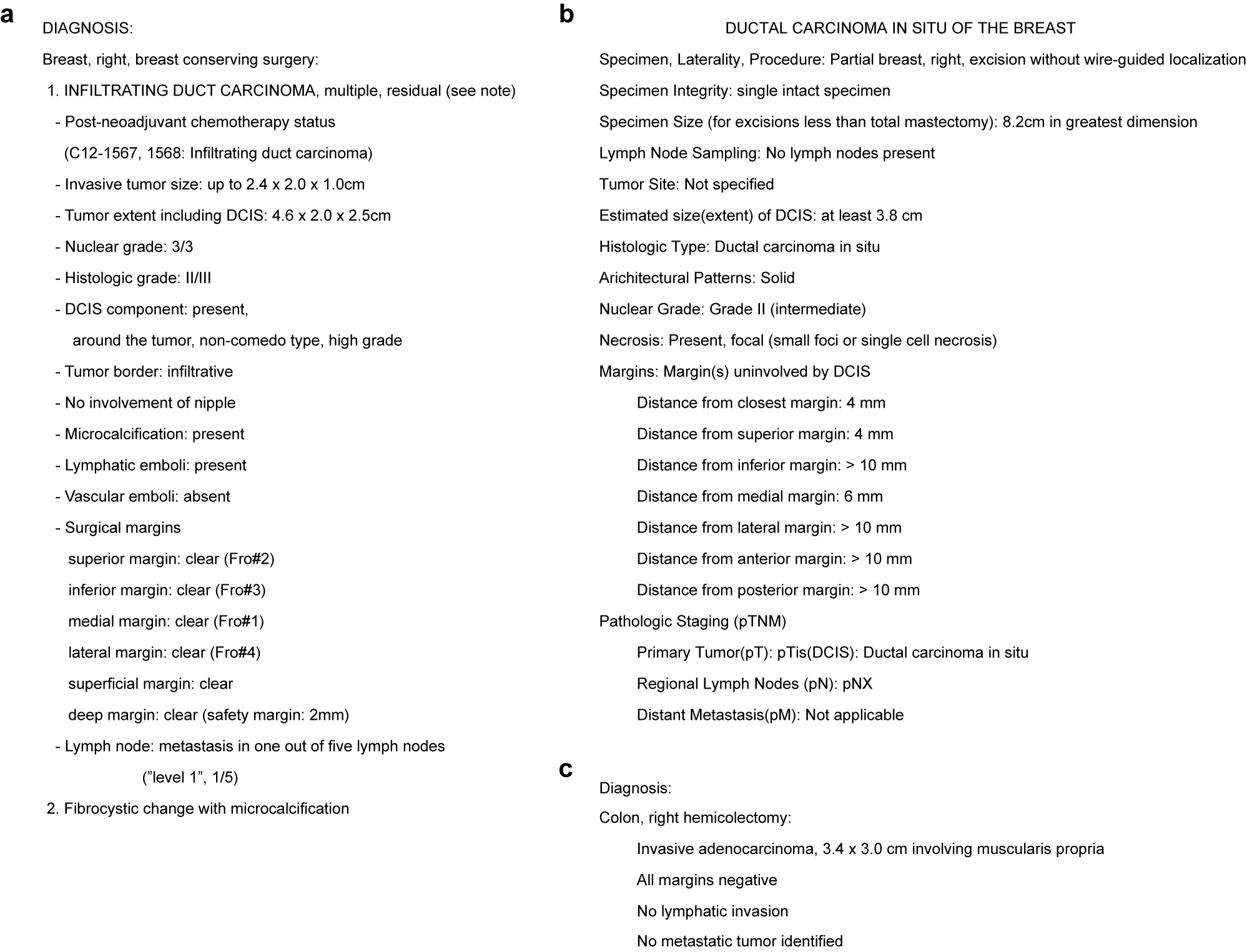

Supplement: Supplementary file 2 — Figure S2. Comparison of SP report styles between SNUH and report templates from CAP. a) SP report at SNUH which shows the combination of synoptic report style and non-synoptic report style. b) synoptic report template for ductal carcinoma in situ of the breast which presents organ information on the first line and describes microscopic findings in the following lines. The synoptic report template shows the grouping of pathologic findings using indentation for better visual separation. c) non-synoptic report template provided by CAP. The first line contains organ information and the second line contains the diagnosis information with indentation for visual separation. (TIF 985 kb) [file 12911_2018_609_MOESM2_ESM.tif]
